# First stage of the Kangoroo Method: adaptation of mothers of premature newborns to Roy’s light

**DOI:** 10.1590/0034-7167-2024-0646

**Published:** 2026-08-07

**Authors:** Adriana Modesto Caxias, Marcia Helena Machado Nascimento, Juliana Conceição Dias Garcez, Danielle Melo Sardinha, Yuri Henrique Andrade de Oliveira, Clareana Costa Campelo, Andressa Tavares Parente, Josias da Costa

**Affiliations:** I Universidade do Estado do Pará. Belém, Pará, Brazil; II Universidade Federal do Pará. Belém, Pará, Brazil

**Keywords:** Kangoroo-Mother Care Method, Intensive Care Units, Neonatal, Premature Birth, Nursing Theory, Nursing Care., Método Madre-Canguro, Unidades de Cuidado Intensivo Neonatal, Recién Nacido Prematuro, Teoría de Enfermería, Atención de Enfermería.

## Abstract

**Objectives:**

to analyze the adaptation process of mothers of premature newborns in the first stage of the Kangoroo Method in light of Callista Roy’s theory.

**Methods:**

a qualitative study that used Callista Roy’s Adaptation Model as a theoretical framework and content analysis according to Bardin. The research took place at a public maternity hospital in July 20254.

**Results:**

fifteen mothers of premature infants participated in the research. The following categories and subcategories emerged: 1) “Stimuli received by mothers of premature newborns”, with the subcategories “Focal stimulus in prematurity settings”, “Contextual stimulus in Neonatal Intensive Care Unit settings” and “Residual stimulus”; 2) “Behavioral responses”, with the subcategories “Physiological”, “Self-concept”, “Role function”, and “Interdependence”.

**Final Considerations:**

participants demonstrated effective adaptation to the context of prematurity and hospitalization. However, ineffective behavioral responses are linked to a lack of care for these mothers, mainly from healthcare professionals.

## RESUMEN

**Objetivos:**

analizar el proceso de adaptación de las madres de recién nacidos prematuros en la primera etapa del

**Método:**

Canguro a la luz de la teoría de Callista Roy.

**Métodos:**

estudio cualitativo que utilizó el Modelo de Adaptación de Callista Roy como marco teórico y el análisis de contenido según Bardin. La investigación se llevó a cabo en una maternidad pública en julio de 2024. R**esultados:** participaron en la investigación 15 madres de bebés prematuros. Se identificaron las siguientes categorías y subcategorías: 1) “Estímulos recebidos por madres de recién nacidos prematuros”, con las subcategorías “Estímulo focal en el contexto de la prematuridad”, “Estímulo contextual en el entorno de la Unidad de Cuidados Intensivos Neonatales” y “Estímulo residual”; 2) “Respuestas conductuales”, con las subcategorías “Modo de función fisiológica”, “Autoconcepto”, “Desempeño de rol” e “Interdependencia”.

**Consideraciones Finales:**

los participantes demonstraron una adaptación eficaz al contexto de la prematuridad y la hospitalización. Sin embargo, las respuestas conductuales ineficaces se relacionan con la falta de atención a estas madres, principalmente por parte de los profesionales de salud.

## INTRODUCTION

Prematurity is defined as a complex clinical syndrome resulting from a process triggered by several factors, determined by birth before 37 completed weeks of gestation^(1,2)^. For every ten newborns worldwide, one is premature, meaning one birth occurs every two seconds^(3)^. In 2020, the number of preterm births worldwide was 13.4 million. In Brazil, in 2021, the incidence of theses births was 302,636, with the rate per region around 11%^(3,4)^.

The occurrence of premature birth can have impacts on newborns’ extrauterine adaptation, considering that various vital systems are still developing. The lower the gestational age, the greater the repercussions on premature infants’ health, with a higher risk of morbidity and mortality. Due to this, a premature newborn (PTNB) is considered high-risk and may require admission to a Neonatal Intensive Care Unit (NICU) after birth^(5)^.

Therefore, hospitalization often becomes a difficult situation for the mothers of theses newborns, who find themselves in a moment of early separation, in addition to the unfamiliar and challenging setting of NICUs. For them, this separation can directly affect the building of the bond between them, as actions such as skin-to-skin contact, breastfeeding, or even touching are often not possible due to newborns’ clinical condition or even a lack of knowledge about the equipment within the unit, which end up causing feelings of insecurity and frustration^(6)^.

Due to concerns regarding all these aspects related to NICUs and neonatal care in general, there was a need to work on technical care in conjunction with humanized care, with the aim of promoting long-term benefits for newborns and their families. Thus, humanization in healthcare has become a necessary and increasingly present practice in neonatal units, considering that it is a set of actions aimed at providing healthcare with welcoming, ethics, and respect for patients’ cultural diversity and uniqueness, as well as spaces with favorable conditions for both professionals and service users^(5,6)^.

In Brazil, to reinforce humanized care for newborns and their families, the Kangaroo Mother Care (KMC) public policy was established through Ordinance 1,683/2007, which advocates for the care of low birth weight newborns (< 2,500 grams) and PTNBs. It is divided into three stages, the first beginning in the prenatal period, also including the NICU and the Conventional Neonatal Intermediate Care Unit. The second stage involves the Kangaroo Mother Care Neonatal Intermediate Care Unit. The third stage includes outpatient and home care^(7)^.

Since its first stage includes the NICU as one of its units, one of the main concerns is facilitating the establishment of a bond between newborns and their families through skin-to-skin contact, with kangaroo care being recommended as early as possible^(8)^. Added to this is the importance of a support network, which includes family and close friends, as well as the healthcare professionals present in the unit, being fundamental for mothers to face the adaptation process to the experienced context and develop their maternal role through the care of their children^(9)^.

That being said, nurses play a very important role, since the activities performed by them and the entire team seek to promote comfort and reduce stress levels in this setting, with the main goal being humanized care for the effective adaptation of newborns, in which mothers also become part of the care provided^(10,11)^.

From this perspective, as a way to provide comprehensive care and promote the well-being of their clients, aiming at their adaptation to the situation in which they find themselves, Callista Roy’s Adaptation Theory stands out, based on the premise that this model can guide nursing care and actions towards this adaptive process^(12)^.

Roy’s Theoretical Model consists of four metaparadigms: the person receiving nursing care, the concept of environment, the concept of health, and the direction of nursing activities. In this study, the person is the mother of a PTNB, who is also the recipient of nursing care and is viewed holistically and adaptively, as she is continuously interacting with her surrounding environment, receiving stimuli and changes from it. For Roy, the environment is not just the physical environment. It consists of all the conditions, circumstances, and influences that surround a person and can affect their development and behavior, which can interfere with their adaptation process^(13,14)^. Health is considered both a state and a process, as a person is and can become whole and integrated, since the lack of the latter corresponds to a lack of health. Nursing objectives consist of the adaptive response of a person, which is influenced by and interacts with the constituent elements of adaptative modes^(12)^.

Stimulus is considered the input, i.e., any interaction between a person (internal stimulus) and an environment (external stimulus) that results in a response (output), where resulting behaviors can be adaptive, promoting a person’s integrity as an adaptable being, or inefficient, where adaptation is not achieved^(15,16)^. Stimuli can be classified in three-ways - focal, contextual, and residual - and may occur simultaneously or not. A focal stimulus is a change or event of an internal or external nature that confronts a person immediately, possessing the greatest impact. Contextual stimuli are called secondary stimuli and are related to the environment and society, influencing, positively or negatively, how a person reacts to the focal stimulus. A residual stimulus may have an indeterminate impact, its influence on a person may or may not be clear, but it still has an effect on the current situation^(16,17)^. Therefore, behaviors resulting from these stimuli can be observed in four adaptive modes: physiological, self-concept, role function, and interdependence^(13)^.

As a basis for supporting and organizing nursing care practice in settings such as the first stage of KMC, theoretical models make it possible to relate concepts to address certain phenomena concerning a person being cared for (whether the individual, the family, or the community), allowing them to be assisted holistically^(11)^.

## OBJECTIVES

To analyze the adaptation process of mothers of preterm newborns in the first stage of KMC in light of Callista Roy’s theory.

## METHODS

### Ethical aspects

This study was approved by the *Universidade do Estado do Pará* and *Fundação Santa Casa de Misericórdia do Pará* Research Ethics Committees. The mothers of PTNBs were approached with a brief explanation of the project and its objectives, and a formal invitation to participate in the study was extended. Upon acceptance, the Informed Consent Form, printed in duplicate, was given to all participants, read, and signed, after which the interview began.

### Study design

This is a qualitative study that used Callista Roy’s Adaptation Model as a theoretical framework. The stages outlined in the COnsolidated criteria for REporting Qualitative research were followed, according to three domains: reflexivity, study concept, and analysis and results^(18)^.

### Study setting

The study was conducted at the *Fundação Santa Casa de Misericórdia do Pará*, a leading maternal and child health hospital located in the municipality of Belém. It has a total of 60 NICU beds, assisting newborns born in the hospital itself or in other healthcare institutions. It is a certified and reference unit in KMC, holding the title of Baby-Friendly Hospital.

### Data source

The sample for the study was purposive^(19)^, and the approach was carried out in person and randomly, with the inclusion criteria being mothers of premature infants (moderately premature, very premature and extremely premature infants), whose children’s stay in the NICU, the first stage of KMC, was > 48 hours. None of the mothers approached presented exclusion criteria, which consisted of mothers who presented altered levels of consciousness and/or of other nationalities. For the interviews, the criterion of response saturation was considered, frequently used in qualitative research, which occurs when responses become similar and there are no situations considered new or divergent that could contribute to the research’s theoretical considerations^(20)^. Thus, a total of 18 mothers were invited, but only 15 participated in the research, meeting the response saturation criterion. The other three mothers did not participate for the following reasons: two accepted, but were not in the ward on the days and times the interviews were conducted; and one declined to participate because she was emotionally fragile when discussing the topic at that time.

### Data collection

A semi-structured interview guide was used for individual interviews, developed specifically for this study, with closedand open-ended questions. Questions arose regarding: focal stimulus (“How did you feel when you received your child’s prematurity diagnosis?” and “How do you currently feel about your child’s prematurity diagnosis?”); contextual stimulus (“How do you feel (or did you feel) about NICU settings?”, “What changed in your routine after your child’s birth?, and “How would you describe your relationship with your family after your child’s birth?”); and residual stimulus (“Have you had any experience related to NICU admission before? If so, what were they?” and “Have you had any experience related to premature birth before? If so, what were they?”). Behavioral responses, according to the four adaptive modes of Callista Roy’s Theory, consisted of questions about: physiological (“During your child’s stay in the NICU, how was(is) your health?”); self-concept (“How do you see yourself after the birth of your child?”); role function (“How is (or was) your experience as a mother in a NICU?); and interdependence (“Do (did) you receive care/guidance during your child’s stay? If so, how did you feel about being cared for/guided?”).

The interviews took place in July 2024, after prior scheduling with the hospital’s neonatology nursing management. They were conducted by the principal researcher, a neonatal assistant nurse and master’s student, which naturally led her to adopt an empathetic and welcoming approach with the mothers interviewed. This was done in conjunction with the use of techniques to conduct the interviews, which took place in a private room at an opportune time, so as not to interfere with mother’s time with their newborns, medical reports, and other unit-related activities. All interviews were recorded using an MP3 voice recorder and had an average duration of 7 minutes and 38 seconds. The alphanumeric codes MPTNB (mother of PTNB), followed by a number starting from 01, were used to protect their anonymity.

### Data organization and analysis

Content analysis was carried out according to Bardin, and followed three fundamental phases^(21)^: 1) Pre-analysis, with intense contact with the material collected in the interviews and the transcription of speeches into individual documents in Microsoft Office Word 2013, totaling 15 documents, named with alphanumeric codes (MPTNB 01, MPTNB 02…); 2) Material exploration and data coding, based on the recording units, creating eight more documents using the same program, each corresponding to the topics of the semi-structured script, named in the following manner and sequence: “focal stimulus”; “contextual stimulus”; “residual stimulus”; “physiological”; “self-concept”; “role function”; “interdependence”; and “coping mechanisms”. Participants’ statements were compiled and organized in each document and in sequence; 3) Processing of results and interpretation, with categorization being carried out, which consists of classifying the elements according to their similarities, with subsequent regrouping according to common characteristics. The transcribed data were categorized and analyzed in light of Callista Roy’s Adaptation Theory, in order to better understand the adaptive processes involved in the first stage of KMC.

## RESULTS

The ages of the 15 interviewees ranged from 19 to 35 years, with a mean of 28 years. Regarding race/color, ten self-identified as brown, three as white, and two as black. Eight participants reside in the Metropolitan Region of Belém, where the hospital is located, while seven reside in other municipalities. After analyzing the interviews, data were organized according to the following thematic categories and subcategories: Category 1 “Stimuli received by mothers of premature newborns”, with the subcategories “Focal stimulus in prematurity settings”, “Contextual stimulus in Neonatal Intensive Care Unit settings” and “Residual stimulus”; Category 2 “Behavioral responses according to Roy’s adaptive modes”, with the subcategories “Physiological”, “Self-concept”, “Role function”, and “Interdependence”.

### Category 1 - Stimuli received by mothers of premature newborns

#### Focal stimulus in prematurity settings

Focal stimulus is identified in the resulting range of diverse feelings generated by the mothers of these babies. Upon receiving the diagnosis of prematurity of their child, various feelings surfaced, such as surprise, sadness, fear of the child’s death, shock, anguish, worry, among others.

*It’s a kind of despair, I don’t know, I can’t explain it to you. Only those who go through this moment of prematurity can truly feel it, you know, the worry, the fear of losing, of not surviving, because that’s the first thing we think, “Am I going to lose my child?”. And then all the despair comes.* (MPTNB 07)

*It was* [...] [voice breaking, tearful] *it was, like, shocking for me, you know, because the doctors said that if I had her soon, she wouldn’t survive. So, I felt really bad.* (MPTNB 01)

*It’s a Shock, because we didn’t expect it either. Besides being premature, it also comes with the problems, you know, that we still have to deal with, but* [...] *it’s difficult.* (MPTNB 14)

Two of the mothers reported that they had already expected the premature birth of their children due to risk factors they presented during pregnancy, leading them to adopt a more relaxed attitude compared to the others.

*When I received the news that the baby might be premature, I kept my faith, I wasn’t afraid at any point; I trusted in God and that He would do what was best for my son, and that I could endure the pregnancy as long as I could.* (MPTNB 05)

*I had problems during my pregnancy with high blood pressure, which was already a risk factor, and I was also taking medication for thrombophilia. So, I had an idea that premature birth could happen, you know, but it wasn’t certain.* (MPTNB 11)

However, as the situation can change, the way of reacting to stimuli can also change, as shown in their statements after their son’s stay in the NICU:

*Sometimes anxiety kicks in, you know? We get worried that something bad will come up in the morning when we get the report card. We hope it doesn’t, but I’m living day by day in relation to it.* (MPTNB 05)

*It’s something that scares us, you know? It’s very challenging because you don’t know exactly what’s going to happen. It’s* [...] *each day is a day, you know. One day at a time. It’s one struggle at a time. Some days are good, others not so much, and that brings us a lot of anxiety.* (MPTNB 11)

Other mothers also report on what it is like to face the prematurity diagnosis after their child’s hospitalization, with a more confident and hopeful discourse, highlighting the importance of skin-to-skin contact, as recommended by KMC:

*Nowadays?*[deep breath]*. Oh, yeah, I felt sad, you know, because* [...] *he had a bad time* [...] *he went back on the machine too* [...] *and lost weight. Now I’m happy, you know, because he’s off the machine, now he’s eating, and he’s gained weight too.* (MPTNB 04)

[...] *so now I’m going to start holding her, you know, carrying her, like I couldn’t before, because she was very premature* [...]*. I couldn’t, you know, but now she’s much better* [...]*. Being able to hold her, just to see her, you know, is already a joy for me, because before she was* [...] *crying, and I, well, didn’t want to know anything. Now I can hold her already.* (MPTNB 06)

#### Contextual stimulus in Neonatal Intensive Care Unit settings

Contextual stimulus is considered an evident stimulus, referring to all environments factors that will influence, positively or negatively, the behavior (or response) triggered by the focal stimulus^(14)^, as occurs in NICU settings:

*Yeah, I got a little worried when I saw it, because I’d never been in an ICU before. I’d never seen what it was like, I’d only heard about it, you know? And when I found myself here inside the ICU, I got a little worried when I saw my daughter all intubated* [...] *well* [...] *like that, you know, breathing with the help of machines.* (MPTNB 01)

*We see the ICU as* [...] *as if it were the end of the tunnel, right? We think of the worst, and* [...] *not today, I feel safer. You know, I already have a sense of what an ICU is like. It’s not the end, you understand. And we can have hope inside the ICU. That it’s one day at a time, but that doesn’t mean we’re going to lose.* (MPTNB 11)

The setting of this unit is built primarily on the relational component, which is directly linked to the staff present, whose role is not only to allow mothers to enter the units, but also to involve them in care and inform them about their children’s clinical condition.

*I feel more at ease. The people, the staff, are attentive. We ask questions. If we have any doubts, they answer them. When I’m* [...] *well, anxious, they treat me with such care and delicacy. They are wonderful.* (MPTNB 08)

*I felt very welcomed the nurses and doctors talked to me well, gave me a lot of information, that they were doing well, that they were recovering.* (MPTNB 12)

*I feel a bit uncomfortable, actually, on weekends, which I think is* [...] *it’s kind of vague, you know. There’s not much information, it’s* [...] *weekends, for me, as a mother, are more painful.* (MPTNB 07)

The changes in this mother’s routine represent an influential factor in her son’s adaptation, as she completely alters her organization and daily life to dedicate herself exclusively to him.

*Everything changed, my routine, I had to move to another city, I had to* [...] *because I want to be close by all the time, so everything changed in my routine. I moved to another city, I moved houses, I changed everything.* (MPTNB 02)

*The thing is, now I have to come to the hospital every day to pump milk, and then I have to express it* [referring to manually expressing breast milk]*. All of this is new to me; it’s changed my routine.* (MPTNB 10)

The family context becomes significantly important for this mother and positively helps in their adaptation, as it represents support amidst the entire situation they are experiencing.

*My family is my support, my support network. Thank God, I have a very large support network, so from the beginning, they all knew that my pregnancy was high-risk. Everyone was there helping me.* (MPTNB 05)

*Today, I see that we’re becoming more and more united, right? That regardless of everything, I have their support, which is the most important thing. So, I saw that she came only to bring more unity to our family and make us even closer.* (MPTNB 02)

#### Residual stimulus

Residual stimuli are generated from internal and external environmental influences, from direct or indirect experiences, and can be indeterminate^(13)^. Two mothers reported having experience with the NICU and prematurity. In the first statement, a mother expressed an unsuccessful experience, influencing the stimulus triggered at the beginning of her second child’s hospitalization. The second mother, despite having experienced the NICU routine, found herself in a new experience with her younger child.

*My daughter. She was in the ICU for 15 days. I went through some very difficult things there, you know. I was very scared at first, because of everything I had already been through. But I see that everything is different now. Everything is different.* (MPTNB 09)

*My 2-year-old daughter also went through the NICU, as she was also born prematurely. So, I already had some experience. But even having that experience, already knowing what it’s like, what it will be like, it’s like you’ve never been there before, like* [...] *it really takes you by surprise.* (MPTNB 14)

In another mother’s statement, the presence of residual negative stimuli is also noticeable, but through indirect experiences:

*I had never, ever been through that before, and when I heard about it, my thought was also that the children wouldn’t be able to survive. Because sometimes we lack information, experience, knowledge, you know, but today I see that* [...] *it’s not quite how I imagined it.* (MPTNB 07)

### Category 2 - Behavioral responses according to Roy’s adaptive modes

#### Physiological

Changes can occur in this mother’s health in various ways, such as in her sleep and rest patterns, which can also result in physical discomfort, in addition to emotional changes such as feelings of sadness, fear, and depression.

*Physically, I still feel pain from my surgery. Of course, I didn’t have adequate rest, and you have to* [...] *sit, stand, the chairs aren’t entirely comfortable. Now, regarding the psychological aspect, I think that, at the moment, I’m much better, because right after everything happened, I was very shaken emotionally, psychologically, I think I became very fragile.* (MPTNB 11)

*Right from the start, I was quite depressed.* [...] *I was very worried about the fact that I had left him here and gone home. For us, it was a shock.* (MPTNB 09)

*Oh, I haven’t stopped to think about myself yet. I still don’t know exactly how I am, neither physically nor psychologically. So, I don’t know.* (MPTNB 08)

*Today I’m coming* [...] *I’m still* [...] *still in postpartum recovery. I’m trying to work on my mental health every day so it doesn’t affect him or me. But* [...] *so far, it’s* [...] *okay. Not one hundred percent, but it’s okay.* (MPTNB 13)

#### Self-concept

The study found evidence of maternal involvement in effective adaptation, with a desire to improve self-care, a willingness towards improved self-concept, and increased hope, from a maternal perspective. However, discoursers influencing negative adaptation were also observed.

*I can’t anymore, I can’t* [...] *be like I used to. I think about my daughter all the time, and* [...] *I already* [...] *I have to take care of myself. My husband says, “You have to eat, you have to feed yourself”. And I’m constantly thinking about* [...] *I have to take care of myself, to take care of her too. That’s it* [laughs]*, I’ve changed completely.* (MPTNB 08)

*I still don’t see myself as a mother, it hasn’t sunk in yet* [slight smile]. (MPTNB 10)

*Oh, I see myself as a different person* [laughs]*. From waking up to going to sleep, my thoughts are on her. On God too, I ask for a lot of prayer, I pray a lot. It’s* [...] *a feeling I’ve never felt before, even when I got pregnant the first time, now with her* [...]*, with a live baby, you know, ah, I have a lot of hope that everything will be alright, God willing.* (MPTNB 15)

*I see myself happier, more* [...] *with expectation of them recovering. More hopeful.* (MPTNB 12)

#### Role function

The role of mothers within the NICU is demonstrated through discourses in various senses, nothing that the meanings change according to the moment experienced.

*It’s been* [...] *well, something new, different* [...] *because I imagined it differently, that I would breastfeed her, you know, in her mouth. So, I have to express milk, go to the Milk Bank, where they bring it and give it to her through a feeding tube. So, it’s very different from how I imagined it.* (MPTNB 10)

*I come every day, but* [...] *it’s been good. I’ve already changed them; I’ve already picked them up.* (MPTNB 14)

*Sometimes I get a little lost, but the girls* [nursing staff] *always help me when I have any questions* [...]*. I’m already very anxious about him being held in my lap, things like that. So, I get a little restless.* (MPTNB 09)

*I’m trying to do my best, you know. I’m trying to be a present mother for him, so he feels my presence there, you know. And I want to pass that peace of mind on to him too, you know.* (MPTNB 05)

#### Interdependence

In the mother’s experience, it is observed that she is determined to change her entire life to spend as much time as possible with her child. However, if she does not feel well supported, her stay may be altered, leading to situations contrary to what KMC guidelines propose for its first stage. This can impact the bonding between the mother and child, the care she provides, and the preparation for a safe discharge, which involves a meticulous process worked on by the NICU team with this mother^(22)^.

*Everyone, my family, my husband’s Family, everyone, my friends, the professionals, I’ve spent a good amount of time here, and I’m already in an environment that I know, so I’m a bit more familiar with people. Even they* [the NICU professionals] *give me strength.* (MPTNB 13)

*She* [the nurse] *instructed me to wash my hands when I arrive, put on a cap, not to enter with hair on. No accessories allowed, to wear a mask, and when touching the baby, we have to use plenty of hand sanitizer to be extra careful, because the maturity is* [...] *is* [...] *extreme prematurity. She requires a litle more* [...] *a little more care.* (MPTNB 03)

*Yes, I’m being taken care of, yes. It’s like I told you, it’s good to be guided, I feel calmer, more* [...] *I feel more informed, and that gives me some relief from the bad thoughts, you know. I* [...] *I feel more relieved.* (MPTNB 08)

*Right now, I’m only receiving assistance regarding the girls, you know. Just regarding them, their recovery, and their care.* (MPTNB 12)

Based on participants’ statements, it is observed that, in premature births, just as newborns are born before the appropriate period, so too is a premature mother born, full of uncertainties, conflicting feelings, and traumas, as this disrupts a unique moment of personal construction and development due to a sudden, unexpected event^(6)^. However, even if the setting usually confronts a person immediately, the way a person responds to stimuli be different. Since settings can change, either suddenly or over a period of time, the way of reacting to stimuli can also change, as they may confront persons in a more insightful way, making responses more evident^(13)^.

## DISCUSSION

The statements of mothers describing their experiences facing a prematurity diagnosis highlight the available stimuli. Changes in feelings are noticeable; while concern, worry, and anxiety persist, feelings of tranquility, strength, and joy for each achievement are also emerging. Touch and skin-to-skin contact have also proven to be positive stimuli, strengthening the bond between mothers and children and influencing mothers to experience positive emotions^(8)^.

Therefore, feelings of hope take shape and become more evident, as does the certainty of living one day at a time, as a way of preparing in case there are any changes in their children’s health, since prematurity refers to a delicate clinical picture that can change suddenly. Due to this, prematurity remains the main setting in which these mothers find themselves. Thus, the focal stimulus is related to the shattering of what was idealized at the time of birth, the diagnosis of prematurity of children, and the feelings surrounding this diagnosis^(13)^.

Regarding contextual support, NICU settings can be quite challenging for these mothers, with the initial impact stemming from a lack of knowledge about its structure and function. Therefore, the Ministry of Health, through KMC, emphasizes the importance of parents becoming familiar with the maternity ward where they plan to give birth, as well as understanding available facilities, including the NICU, and participating in talks with professionals and families who have experienced hospitalization in the unit. This recommendation aims to make parents feel more secure and more accepting should their children’s hospitalization be necessary after birth, consequently reducing anxiety^(17-23)^.

As also highlighted by KMC, the presence of a support network offering affection, support, and empathy towards mothers’ situation signifies protection in relation to the physical separation between them, their children, and their families, as well as providing emotional support to help them cope with this difficult time of change. Furthermore, it contributes to developing their maternal role more effectively^(8)^.

It was observed that the residual stimuli presented by participants initially influenced their behavior, even unconsciously. However, these stimuli underwent modifications with the current experiences of these women, modifications triggered by the focal stimulus (change in perception regarding the diagnosis of prematurity of their current child) and contextual stimulus (physical and relational environment in the NICU and presence of a support network), which stimulated a favorable adaptation framework^(13)^.

The response in physiological is associated with physical and physiological manifestations of the organism, and can also alter a person’s emotional behavior. Experiencing negative feelings regarding prematurity and their children’s hospitalization in a NICU is almost inevitable for this mother. The postpartum period, which already presents changes in mothers’ lives, naturally ends up being more compromised than expected, since there is an alteration in their sleep and rest pattern due to their frequent trips to the NICU to be with their children. Thus, postpartum women, who should have a period of rest to recover from the entire process of pre-partum, delivery, and postpartum, whether after a cesarean section - where recovery is slower - or after a vaginal delivery, begin to see their health as a lesser priority at that moment, experiencing greater fatigue than usual^(11,13)^.

Nevertheless, it is recommended that professionals involved in care, particularly those in the nursing field - who continuously monitor the mother-child dyad - always consider and pay attention to mothers’ responses, as this will allow them to intervene through actions aimed at reducing any unfavorable responses they may exhibit^(14-24)^. Considering the context of KMC, it is also crucial that healthcare professionals facilitate contact between mother and child as early as possible, as this influence has shown positive results in stabilizing physiological symptoms in both mother and baby, reducing maternal stress levels, and increasing the bond between them^(8-24)^.

In self-concept, which deals with the basic need for a person’s psychic integrity, referring to their psychological and spiritual aspects, attachment to spirituality stood out as a source of strength for these mothers. It is also important to emphasize that adaptation problems in this mode can interfere with a person’s ability to heal or even to perform the minimum care necessary to maintain their health^(13)^. In a study conducted with six mothers of PTNBs admitted to a Baby-Friendly Hospital, participants reported that the abrupt birth of their children and subsequent admission to the NICU somehow impaired their psychological understanding of this new perspective on themselves, motherhood, and their child^(25)^.

The role of mothers is directly related to her physical presence in NICUs, since according to KMC, mothers are not visitors, but rather companions to their babies, even if a baby is in intensive care^(8)^. This approach resolves around what a person has or aspires to for themselves. Adaptation to this new role will depend on how they face the changes before them, reflected in the context of prematurity and NICU settings, which can lead to the risk of mothers distancing themselves from their children’s care, culminating in a setting where this care ends up being provided entirely by healthcare professionals. For this reason, it is essential that this professional assists and guides mothers regarding newborn care and in recognizing warning signs, considering that they will be the one caring for children after hospitalization^(5,24)^.

The performance of routine activities, such as holding children and breastfeeding, which are natural characteristics of the maternal role, ends up undergoing alterations, leading to the delayed attainment of the maternal role, resulting from limited contact and interaction between mother and child^(11)^. In a study conducted in NICUS in four Brazilian states, ten women who had premature births were interviewed about their experience during the first week of their newborns’ hospitalization. They reported fear of physical contact with their newborns, perceiving it as a fragile and very small being surrounded by devices (orotracheal tube, orogastric tube, central catheter, among others), and that handling it could cause the disconnection of one of these devices, representing a barrier to physical contact and limiting essential care such as diaper changing^(26)^.

However, throughout hospitalization, mothers present in the study’s NICU begin to perceive the various ways of providing care to their children, considering their specific needs, and of being closer to and connecting with them, using, for instance, the kangaroo position, through daily learning^(24)^.

In interdependence, support systems are configured through interpersonal relationships, usually occurring with people close to individuals, where emotional needs are met in a setting where caregivers are also cared for^(14)^. In a study conducted, the mothers interviewed mentioned feeling cared for by the NICU team, and observed the effort put into caring for their children, which contributes to neonates’ clinical progress. The importance of instructing them about the equipment surrounding newborns is also clear, as it is essential for their survival, but without adequate explanation of its purpose and function, it ends up representing barriers to contact between the mother and baby^(5)^.

Considering that Roy states that the present mode has a sense of security as its main basic need to nurture various types of relationships, effective adaptation occurs through relationships of this nature, which, as a result, encourage other positive feelings that can contribute to mothers’ adaptation to their reality^(13)^. Therefore, interpersonal relationships, depending on the context and their progress, can facilitate or hinder the adaptation process. The presence of clear and open dialogue between healthcare professionals and mothers in NICUs fosters a beneficial relationship, in which mothers feel safe, supported, respected, and valued as a result of this interaction^(5,11)^.

### Study limitations

Study limitations refers to the fact that it was conducted with mothers of preterm infants admitted to the NICUs of the same healthcare service, as this may have contributed to similarities in participants’ experiences. Conducting the study in other health institutions could lead to a greater possibility of finding different experiences and narratives.

### Contributions to nursing, health, or public policy

This study may contribute to the care provided by nurses in promoting the adaptation of mothers of preterm infants during their stay in the NICU, based on knowledge of the experiences lived by these mothers, in order to minimize the anxiety and concerns they may have, as well as the fear about the unknown due to their children’s hospitalization so soon after birth. It may also encourage future studies on the subject.

## FINAL CONSIDERATIONS

It was possible to conclude that the participants showed effective adaptation after a period of their child’s hospitalization. However, as advocated by the first stage of KMC, such adaptation needs to occur as early as possible. It was inferred that the ineffective behavioral responses observed are mostly linked to the lack of care for these mothers, mainly by NICU healthcare professionals, who are fundamental actors in guaranteeing comprehensive and qualified care according to the KMC policy, as well as in the adaptation process of these mothers.

The use of Roy’s Theory allowed us to recognize that nursing care should also encompass mothers, who are not always prioritized, but needs to adapt to a reality that was previously completely unknown to them, filled with fear and insecurity about their children’s future, which can lead them to present a positive or negative response. It is important that the nurse constantly assesses and intervenes with this mother in order to alter stimuli that may harm their well-being and, consequently, their adaptation to environments.

## Data Availability

The research data are available within the article.
