# Supplementary material for: First stage of the Kangoroo Method: adaptation of mothers of premature newborns to Roy’s light
Source: Rev Bras Enferm. 2026 Aug 7;79:e20240646. doi: 10.1590/0034-7167-2024-0646 (PMC13450832; doi:10.1590/0034-7167-2024-0646)
Supplement: Translation

# Translation: Primeira etapa do Método Canguru: adaptação de mães de recém-nascidos prematuros à luz de Roy

**DOI:** 10.1590/0034-7167-2024-0646pt

**Authors:** Adriana Modesto Caxias, Marcia Helena Machado Nascimento, Juliana Conceição Dias Garcez, Danielle Melo Sardinha, Yuri Henrique Andrade de Oliveira, Clareana Costa Campelo, Andressa Tavares Parente, Josias da Costa

**Affiliations:** I Universidade do Estado do Pará. Belém, Pará, Brasil; II Universidade Federal do Pará. Belém, Pará, Brasil

**Keywords:** Método Canguru, Unidade de Terapia Intensiva Neonatal, Recém-Nascido Prematuro, Teoria de Enfermagem, Cuidado de Enfermagem.

## Abstract

**Objetivos:**

analisar o processo de adaptação das mães de recém-nascidos prematuros na primeira etapa do

**Método:**

Canguru à luz da teoria de Callista Roy.

**Métodos:**

estudo qualitativo, que utilizou como referencial teórico o Modelo de Adaptação de Callista Roy e análise de conteúdo segundo Bardin. A pesquisa aconteceu em uma maternidade pública em julho de 2024.

**Resultados:**

participaram da pesquisa 15 mães de prematuros. Emergiram as seguintes categorias e subcategorias: 1) “Estímulos recebidos pelas mães dos recém-nascidos prematuros”, com as subcategorias “Estímulo focal no cenário da prematuridade”, “Estímulo contextual no ambiente da Unidade de Terapia Intensiva Neonatal” e “Estímulo Residual”; 2) “Respostas comportamentais”, com as subcategorias “Modo função fisiológica”, “Autoconceito”, “Desempenho de papéis” e “Interdependência”.

**Considerações Finais:**

as participantes apresentaram adaptação eficaz ao contexto da prematuridade e da internação. Contudo, as respostas comportamentais ineficazes estão ligadas à ausência de cuidados com essa mãe, principalmente pelos profissionais de saúde.

## INTRODUÇÃO

A prematuridade é definida como uma síndrome clínica complexa resultante de um processo desencadeado por vários fatores, determinada com o nascimento antes das 37 semanas completas de gestação^(1,2)^. A cada dez recém-nascidos (RNs) que nascem no mundo todo, um é prematuro, o que leva a um nascimento dessa natureza a cada dois segundos^(3)^. Em 2020, o número de partos pré-termos foi de 13,4 milhões no mundo todo. No Brasil, em 2021, a incidência desses partos foi de 302.636, sendo a taxa por Região em torno de 11%^(3,4)^.

A ocorrência desse parto precoce pode apresentar impactos na adaptação extra útero do RN, considerando que os mais diversos sistemas vitais ainda estão em desenvolvimento. Quanto menor a idade gestacional, maiores são as repercussões na saúde desse prematuro, havendo maior risco para morbimortalidade. Devido a isso, o recém-nascido prematuro (RNPT) é tido como de alto risco, podendo necessitar de internação em Unidade de Terapia Intensiva Neonatal (UTIN) após o nascimento^(5)^.

Logo, a internação se torna uma situação muitas vezes difícil de lidar para as mães desses neonatos, que se descobrem em um momento de separação precoce, além do ambiente desconhecido e desafiador da UTIN. Para ela, esta separação pode afetar diretamente na construção do vínculo entre ambos, pois ações como contato pele a pele, amamentação ou mesmo toque muitas vezes não são possíveis devido ao quadro clínico do RN ou mesmo ao desconhecimento acerca dos equipamentos dentro da unidade, que acabam acarretando sentimentos de insegurança e frustração^(6)^.

Por conta da preocupação acerca de todos esses aspectos relacionados à UTIN e ao cuidado neonatal de forma geral, houve uma necessidade de trabalhar o cuidado técnico em consonância com o cuidado humanizado, com o intuito de promover benefícios a longo prazo para o RN e sua família. Assim, a humanização em saúde se tornou uma prática necessária e cada vez mais presente nas unidades neonatais, tendo em vista que se trata de um conjunto de ações que visam ao cuidado em saúde com acolhimento, ética e respeito à diversidade cultural e singularidade do paciente, além de espaços com condições favoráveis para o profissional e o usuário dos serviços^(5,6)^.

No Brasil, para reforçar a atenção humanizada ao RN e sua família, foi instituída a política pública do Método Canguru (MC), através da Portaria nº 1.683/2007, a qual preconiza o cuidado ao RN de baixo peso (< 2.500 gramas) e RNPT. É dividida em três etapas, em que a primeira inicia no pré-natal, fazendo parte também a UTIN e a Unidade de Cuidados Intermediários Neonatal Convencional. Na segunda, tem-se a Unidade de Cuidados Intermediários Neonatal Canguru; e na terceira, os cuidados ambulatoriais e domiciliares^(7)^.

Por sua primeira etapa ter como uma das unidades a UTIN, uma das principais preocupações é facilitar o estabelecimento do vínculo entre o RN e sua família, através do contato pele a pele, sendo recomendada a posição canguru o mais precoce possível^(8)^. Somam-se a importância da rede de apoio, que engloba familiares e pessoas próximas, bem como os profissionais de saúde presentes na unidade, sendo fundamentais para que a mãe possa enfrentar o processo de adaptação ao contexto vivenciado e desenvolver seu papel materno através dos cuidados com seu filho^(9)^.

Isto posto, o enfermeiro possui papel de grande importância, visto que as atividades desempenhadas por ele e por toda a equipe buscam promover a oferta do conforto e diminuição do nível de estresse nesse ambiente, tendo como principal meta a assistência humanizada para adaptação eficaz do RN, em que a mãe também passa a fazer parte dos cuidados prestados^(10,11)^.

Nessa perspectiva, como forma de prestar assistência integral e promover o bem-estar de seus clientes, visando sua adaptação à situação em que se encontram, destaca-se a Teoria da Adaptação de Callista Roy, partindo da premissa de que esse modelo pode conduzir os cuidados e ações de enfermagem a esse processo adaptativo^(12)^.

O Modelo Teórico de Roy consiste em quatro metaparadigmas: a pessoa que recebe o cuidado de enfermagem, o conceito de ambiente, o conceito de saúde e a direção das atividades de enfermagem. Neste estudo, a pessoa consiste na mãe do RNPT, sendo ela também a receptora dos cuidados de enfermagem e vista de forma holística e adaptativa, pois encontra-se interagindo com o ambiente que a cerca continuamente, recebendo os estímulos desse ambiente e suas mudanças. O ambiente, para Roy, não se trata apenas do ambiente físico. Ele consiste em todas as condições, circunstâncias e influências que cercam a pessoa e podem afetar seu desenvolvimento e comportamento, o que pode interferir no seu processo de adaptação^(13,14)^. A saúde é tida como um estado e, ao mesmo tempo, um processo, pois a pessoa é e pode se tornar total e integrada, visto que a falta desse último corresponde à falta de saúde. Os objetivos da enfermagem consistem na resposta adaptativa da pessoa, sendo esta influenciada e em interação com os elementos constituintes dos modos adaptativos^(12)^.

O estímulo é tido como a entrada, ou seja, qualquer interação entre a pessoa (estímulo interno) e o ambiente (estímulo externo) que resulta em uma resposta (saída), em que os comportamentos resultantes podem ser adaptativos, promovendo a integridade da pessoa como ser adaptável, ou ineficientes, em que não há o alcance da adaptação^(15,16)^. Podem ser classificados de três formas - focal, contextual e residual -, e podem acontecer paralelamente ou não. O estímulo focal é uma mudança ou um evento de natureza interna ou externa que confronta a pessoa de forma imediata, possuindo o estímulo de maior impacto. Os estímulos contextuais são chamados de secundários, e estão relacionados ao ambiente e sociedade, influenciando, de forma positiva ou negativa, a maneira como a pessoa age ao estímulo focal. O estímulo residual pode possuir impacto indeterminado, podendo estar clara ou não sua influência sobre a pessoa, porém, ainda assim, com efeito na situação atual^(16,17)^. Logo, os comportamentos resultantes desses estímulos podem ser observados em quatro modos adaptativos: modo função fisiológica, autoconceito, desempenho de papéis e interdependência^(13)^.

Como base para sustentar e organizar a prática assistencial de enfermagem em cenários como a primeira etapa do MC, os modelos teóricos possibilitam relacionar conceitos para enfrentar determinados fenômenos acerca da pessoa cuidada (seja o indivíduo, a família ou a comunidade), permitindo que ela seja assistida de forma holística^(11)^.

## OBJETIVOS

Analisar o processo de adaptação das mães de RNPTs na primeira etapa do MC à luz da Teoria de Callista Roy.

## MÉTODOS

### Aspectos Éticos

Este estudo foi aprovado pelos Comitês de Ética em Pesquisa da Universidade do Estado do Pará e da Fundação Santa Casa de Misericórdia do Pará. Foram realizadas a abordagem às mães dos RNPT’s, com uma breve explicação sobre o projeto e seus objetivos, e a formalização do convite para participar do estudo. Ao aceitar, foi entregue o Termo de Consentimento Livre e Esclarecido, impresso em duas vias, sendo lido e assinado por todos, e após isso, deu-se início a entrevista.

### Tipo de Estudo

Trata-se de estudo qualitativo, que utilizou como referencial teórico o Modelo de Adaptação de Callista Roy. Foram cumpridas as etapas do guia *COnsolidated criteria For REporting Qualitative researsch*, segundo três domínios: reflexividade, o conceito do estudo e análise e resultados^(18)^.

### Cenário do Estudo

O estudo foi realizado na Fundação Santa Casa de Misericórdia do Pará, um hospital de referência materno-infantil localizado no município de Belém. Possui um total de 60 leitos de UTINs, que atendem RNs nascidos no próprio hospital ou em outras instituições de saúde. É unidade certificada e de referência em MC, possuindo o título de Hospital Amigo da Criança.

### Fonte de dados

A amostra para o estudo foi proposital^(19)^, e a abordagem foi feita de forma presencial e aleatória, tendo como critérios de inclusão mães de prematuros (RNPTs moderados, muito prematuros e RNPTs extremos), cujo período de internação dos filhos na UTIN, primeira etapa do MC, fosse > a 48 horas. Nenhuma das mães abordadas apresentou critério para exclusão, que consistiu em mães que apresentassem nível de consciência alterado e/ou de outras nacionalidades. Foi considerado, para as entrevistas, o critério de saturação de respostas, utilizado com frequência na pesquisa qualitativa, que ocorre quando as respostas se tornam semelhantes e não há situações consideradas novas ou divergentes que possam contribuir com as considerações teóricas da pesquisa^(20)^. Dessa forma, foram convidadas, ao todo, 18 mães, porém, participaram da pesquisa 15, as quais corresponderam ao critério de saturação de respostas. As outras três mães não participaram pelos seguintes motivos: duas aceitaram, porém não se encontravam na ala nos dias e horários em que as entrevistas foram realizadas; e uma não aceitou participar por estar emocionalmente fragilizada ao falar sobre a temática naquele momento.

### Coleta dos Dados

Foi utilizado roteiro semiestruturado para entrevista individual, elaborado para o presente estudo com perguntas fechadas e abertas. Surgiram perguntas sobre: estímulo focal (“Como você se sentiu quando recebeu o diagnóstico de prematuridade do seu filho?” e “Como você se sente atualmente em relação ao diagnóstico de prematuridade do seu filho?”); estímulo contextual (“Como você se sente (ou sentiu) em relação ao ambiente da UTIN?”, “O que mudou na sua rotina após o nascimento do seu filho?” e “Como você descreve o relacionamento com a sua família após o nascimento do seu filho?”); e estímulo residual (“Você já havia passado por alguma experiência relacionada à internação na UTIN antes? Se sim, quais?” e “Você já havia passado por alguma experiência relacionada ao nascimento prematuro antes? Se sim, qual(is)?”). As respostas comportamentais, segundo os quatro modos adaptativos da Teoria de Callista Roy, consistiram nas questões sobre: modo função fisiológica (“Durante a internação do seu filho na UTIN, como esteve(está) sua saúde?”); autoconceito (“Como você se vê após o nascimento do seu filho?”); desempenho de papéis (“Como está sendo (ou foi) sua experiência como mãe dentro de uma UTIN?”); e interdependência (“Você recebe (ou recebeu) cuidados/orientações durante a internação do seu filho? Se sim, como você se sentiu em relação a ser cuidada/orientada?”).

As entrevistas ocorreram, em julho de 2024, após agendamento prévio com a gerência de enfermagem de neonatologia do hospital. Foram realizadas pela pesquisadora principal, que é enfermeira assistente neonatal e mestranda, levando-a a adotar, naturalmente, postura empática e acolhedora com as mães abordadas, concomitante ao uso de técnicas para realizar as entrevistas, que ocorreram em uma sala reservada, em momento oportuno, não prejudicando os períodos das mães junto ao neonato, boletins médicos e demais ações relacionadas à unidade. Todas as entrevistas foram registradas com auxílio de um gravador de voz do tipo mp3, e tiveram uma duração média de 7 minutos e 38 segundos. Foram utilizados os códigos alfanuméricos MRNPT (Mãe de Recém-Nascido Pré-Termo), seguidas de numeração, a partir de 01, como forma de preservar suas identidades.

### Organização e Análise dos dados

A análise do conteúdo foi realizada segundo Bardin, e seguiu três fases fundamentais^(21)^: 1) Pré-análise, com contato intenso com o material coletado nas entrevistas e a transcrição das falas em documentos individuais no programa *Microsoft Office Word* 2013, totalizando 15 documentos, nomeados com os códigos alfanuméricos (MRNPT 01, MRNPT 02, ...); 2) Exploração do material e codificação dos dados, a partir das unidades de registro, sendo criados mais oito documentos utilizando o mesmo programa, em que cada um correspondeu aos tópicos do roteiro semiestruturado, nomeados da seguinte maneira e sequência: “estímulo focal”; “estímulo contextual”; “estímulo residual”; “modo função fisiológica”; “autoconceito”; “desempenho de papéis”; “interdependência”; e “mecanismos de enfrentamento”. As falas das participantes foram compiladas e organizadas em cada documento e em sequência; 3) Tratamento dos resultados e interpretação, sendo realizada a categorização, a qual consiste na classificação dos elementos segundo suas semelhanças, com posterior reagrupamento conforme as características comuns. Os dados transcritos foram categorizados e analisados à luz da Teoria da Adaptação de Callista Roy, com a finalidade de melhor compreender os processos adaptativos envolvidos na primeira etapa do MC.

## RESULTADOS

A idade das 15 entrevistadas variou de 19 a 35 anos, com média de 28 anos. Quanto à raça/cor, dez se autodeclararam pardas, três, brancas e duas, pretas. Residem na Região Metropolitana de Belém, onde está localizado o hospital, oito participantes, enquanto sete residem em outros municípios. Após a análise das entrevistas, os dados foram organizados de acordo com as seguintes categorias e subcategorias temáticas: Categoria 1 “Estímulos recebidos pelas mães dos recém-nascidos prematuros”, com as subcategorias: “Estímulo focal no cenário da prematuridade”, “Estímulo contextual no ambiente da Unidade de Terapia Intensiva Neonatal” e “Estímulo residual”; Categoria 2 “Respostas comportamentais segundo os Modos Adaptativos de Roy”, com as subcategorias “Modo função fisiológica”, “Autoconceito”, “Desempenho de papéis” e “Interdependência”.

### Categoria 1 - Estímulos recebidos pelas mães dos recém-nascidos prematuros

#### Estímulo focal no cenário da prematuridade

O estímulo focal é identificado no consequente quadro de sentimentos diversos gerados pelas mães desses bebês. Ao receber o diagnóstico de prematuridade do filho, sentimentos diversos vieram à tona, como surpresa, tristeza, medo da morte do filho, choque, angústia, preocupação, entre outros.

*Um desespero, assim, eu não sei, eu não sei te explicar. Só quem passa por esse momento de prematuridade é que consegue realmente sentir, sabe, a preocupação, o medo de perder, de não sobreviver, porque é logo o que a gente pensa: “Será que eu vou perder meu filho?”. E vem todo o desespero.* (MRNPT 07)

*Foi* [...] [voz embargada, chorosa] *foi, assim, chocante pra mim, né, porque os médicos diziam que se eu tivesse logo ela não ia sobreviver. Aí eu me sentia bem mal.* (MRNPT 01)

*É um baque, porque a gente não espera também. Fora queee* [...] *além de ser prematuro, aindaa* [...] *comé* [...] *ainda vem com os problemas, né, que a gente ainda tem que, que lidar. Mas* [...] *é difícil.* (MRNPT 14)

Duas das mães relataram já esperar o nascimento prematuro de seus filhos, devido a fatores de risco que apresentavam durante a gestação, levando-as a adotar uma postura mais tranquila em relação às demais.

*Quando eu recebi a notícia de que poderia vim prematuro, né, eu mantive a minha fé, né. Não fiquei com medo em nenhum momento, confiei em Deus e que ele faria o melhor pro meu filho, né, e que eu aguentasse a gestação até onde* [...] *desse.* (MRNPT 05)

*Eu tive problemas na gestação com pressão alta, que já era um fator de risco, e eu também fazia medicação pra trombofilia. Então, eu tinha uma noção que poderia acontecer a prematuridade, sabe, mas não tava definido.* (MRNPT 11)

Contudo, como o cenário pode sofrer mudança, a forma de reagir aos estímulos também pode mudar, conforme mostram suas falas após um período de internação do filho na UTIN:

*Às vezes, bate a ansiedade, né. A gente fica apreensivo de achar que, de manhã, quando vai receber o boletim, vai aparecer alguma coisa. A gente torce que não, né, mas eu tô vivendo dia após dias em relação a isso.* (MRNPT 05)

*É algo, assim, que nos traz medo, né. É muito desafiador, porque você não sabe que, que exatamente vai acontecer. É* [...] *cada dia é um dia, né. Um dia de cada vez. É uma luta de cada vez. Uns dias são bons, outros nem tanto, e aí isso nos traz muita aflição.* (MRNPT 11)

Outras mães também relatam como está sendo enfrentar o diagnóstico da prematuridade após um período de internação do filho, com um discurso mais confiante e permeado de esperança, trazendo à tona a importância do contato pele a pele, recomendado pelo MC:

*Hoje em dia?* [Inspiração profunda]*. Ah, é, eu me senti trise já, né, por causa que* [...] *ele passou ruuim* [...] *ele voltou pro aparelho também* [...] *e perdeu peso. Agora eu tô feliz, né, porque ele saiu do aparelho, agora tá se alimentando, ganhou peso também.* (MRNPT 04)

[...] *aí eu já vô começá a pegá ela, entendeu, carregá ela, como eu num carregava antes, que era bem prematura* [...]*. Num podia, né, aí agora ela tá mais melhor* [...]*. Podê eu carregar ela, assim, pra vê ela, tendeu, já é uma alegria pra mim, porque antes era uma* [...] *chorava, e eu, é, num queria saber de nada. Agora eu já posso já pegá ela.* (MRNPT 06)

#### Estímulo contextual no ambiente da Unidade de Terapia Intensiva Neonatal

O estímulo contextual é tido como um estímulo evidente e refere-se a todos os fatores do ambiente que irão influenciar, de forma positiva ou não, para o comportamento (ou resposta) desencadeado pelo estímulo focal^(14)^, como ocorre no ambiente da UTIN.

*É, fiquei um pouco preocupada quando eu vi, porque eu nunca tinha entrado numa UTI, nunca tinha visto como era, só ouvia falar, né? E quando eu me vi aqui dentro da UTI, eu fiquei um pouco preocupada quando eu vi minha filha toda intubada,* [...] *é* [...] *daquele jeito, né, respirando por aparelhos.* (MRNPT 01)

*A gente vê a UTI como* [...] *como se fosse o fim do túnel, né. A gente pensa no pior, eee* [...] *hoje não, me sinto mais segura. Sabe, já, eu já tenho, assim, uma, uma noção do que é uma UTI. Não é o fim, entendeu. E a gente pode ter esperança dentro da UTI. Que é um dia de cada vez, mas que não significa que a gente vai perder.* (MRNPT 11)

O ambiente dessa unidade se constrói principalmente com o componente relacional, que está diretamente ligada à equipe presente, que tem como papel não somente permitir a entrada da mãe nas unidades, mas envolvê-la nos cuidados e informá-la sobre o quadro clínico do seu filho.

*Eu fico mais tranquila. As pessoas, os funcionários são atenciosos. A gente pergunta alguma coisa. Tendo alguma dúvida, eles tiram as nossas dúvidas. Quando eu tô* [...] *assim, angustiada, tratam com toda, com toda delicadeza. São maravilhosos.* (MRNPT 08)

*Me senti bem acolhida, os enfermeiros, os médicos conversam bem comigo, me dão muitas informações, que elas estão bem, estão se recuperando.* (MRNPT 12)

*Eu sinto um pouco de desconforto, na verdade, assim, nos finais de semana, que eu acho que é uma coi* [...] *fica uma coisa meio vaga, assim, sabe. Num tem muita informação, é* [...] *os finais de semana, pra mim, como mãe, é mais doloroso.* (MRNPT 07)

As mudanças na rotina dessa mãe representam um fator de influência sobre a adaptação, pois ela muda sua organização e seu cotidiano totalmente para se dedicar de forma exclusiva ao seu filho.

*Mudou tudo, minha rotina, tive que mudar de cidade, tive que* [...] *porque eu quero ficar todo tempo perto, então mudou tudo na minha rotina. Mudei de cidade, mudei de casa, mudei de tudo.* (MRNPT 02)

*É que agora eu tenho que vim todo dia pro hospital, tirá leite, aí eu tenho que espremer* [referindo-se à ordenha manual do leite materno]*. Tudo isso é novidade pra mim, mudou a minha rotina.* (MRNPT 10)

O contexto familiar se torna significativamente importante para essa mãe e ajuda de forma positiva na sua adaptação, pois representa apoio em meio a toda situação vivenciada.

*A minha família é o meu apoio, minha rede de apoio. Graças a Deus eu tenho uma rede de apoio muito grande, então desde o início, todos eles descobriram que a minha gravidez era de alto risco. Todo mundo tava ali, me ajudando.* (MRNPT 05)

*Hoje, eu vejo que a gente tá cada vez mais unido, né? Que independente de tudo, eu tenho o apoio deles, que é o mais importante. Então, eu vi que ela veio só pra trazer mais união pra nossa família e deixar a gente cada vez mais perto.* (MRNPT 02)

#### Estímulo residual

O estímulo residual é gerado a partir de influências ambientais internas e externas de vivências diretas ou indiretas, podendo ser indeterminado^(13)^. Duas mães relataram ter experiência com a UTIN e a prematuridade. Na primeira fala, a mãe expressa uma experiência não exitosa, influenciando o estímulo desencadeado no início da internação do segundo filho. Já a segunda, apesar de ter vivenciado a rotina da UTIN, encontrou-se em uma nova experiência com seu filho mais novo.

*A minha filha. Ela ficou 15 dias internada na UTI. Eu passei com coisas muito difíceis lá, né. Eu fiquei com muito medo no começo, por tudo que eu já tinha passado. Mas eu vi que tá sendo tudo. Tudo tá sendo diferente.* (MRNPT 09)

*A minha filha de 2 anos, ela passou também pela UTI, que ela nasceu prematura também. Aí eu já tinha uma experiência. Só que, mesmo já tendo uma experiência, já sabendo, assim, como é, como vai ser, é como se nunca tivesse, como see* [...] *te pegassem mesmo de surpresa.* (MRNPT 14)

Na fala de outra mãe percebe-se também a presença de estímulo residual negativo, mas por vivências indiretas:

*Eu nunca, nunca tinha passado e quando eu ouvia fala. O meu pensamento era esse também, que as crianças não conseguiam sobreviver. Assim, porque a gente, às vezes, é falta de informação, de experiência, conhecimento, né, mas hoje eu vejo que* [...] *que não é bem como eu imaginava.* (MRNPT 07)

### Categoria 2 - Respostas comportamentais, segundo os modos adaptativos de Roy

#### Modo função fisiológica

Alterações podem ocorrer com a saúde dessa mãe de diversas maneiras, como no padrão de sono e repouso, que também têm como consequência desconforto físico, somado a alterações de nível emocional, como sensação de tristeza, de medo e de depressão.

*Fisicamente, eu ainda sinto dores na minha cirurgia. Claro que eu não tive um repouso adequado, e a gente tem que* [...] *senta, levanta, as cadeiras não são confortáveis totalmente. Agora a questão psicológica, eu acho que, no momento, eu tô bem melhor, porque logo quando eu, quando aconteceu tudo e tal, eu ficava bem abalada emocionalmente, psicologicamente, eu acho que eu fiquei muito fragilizada.* (MRNPT 11)

*Logo no começo, eu tava bem depressiva.* [...] e*u tava muito preocupada do fato deu ter deixado ele aqui e ter ido pra casa. Pra gente foi um choque.* (MRNPT 09)

*Ai, eu não parei ainda pra pensar em mim. Eu ainda não sei exatamente como eu tô, nem fisicamente, nem psicologicamente. Então, eu não sei.* (MRNPT 08)

*Hoje eu tô vindo* [...] *ainda tô de* [...] *no resguardo ainda. O psicológico tô tentando trabalhar todo dia praaa não afetar ele e a mim. Mas* [...] *até então tá* [...] *bem. Não cem por cento, mas tá bem.* (MRNPT 13)

#### Autoconceito

Foram encontrados um envolvimento pelas mães para uma adaptação eficaz, com desejo de melhorar o autocuidado, e disposição para autoconceito melhorado e esperança aumentada, a partir de uma perspectiva materna. Todavia, também foram observados discursos com influência para uma adaptação negativa.

*Eu já não, não consigo mais* [...] *sê como antes. Eu penso todo tempo na minha filha, e* [...] *Eu já* [...] *eu tenho que cuidar de mim. Meu marido fala: “Tem que comê, tem que se alimentá”. E eu fico toda hora pensando em* [...] *eu tenho que cuidá de mim, pra cuidá dela também. É isso* [risos]*, eu mudei completamente.* (MRNPT 08)

*Ainda não me vejo como mãe, que ainda não caiu a ficha* [leve sorriso]. (MRNPT 10)

*Ai, me vejo outra pessoa* [risos]*. É do acordá ao dormí, meu pensamento é nela. Em Deus também, eu peço muita oração, eu oro muito. É* [...] *um sentimento que eu nunca senti antes, mesmo quando eu engravidei na primeira vez, agora com ela* [...] *com um bebê vivo, né, ah, eu tenho muita esperança que vai dá tudo certo, se Deus quisé.* (MRNPT 15)

*Me vejo mais alegre, mais* [...] *com expectativa delas se recuperarem. Mais esperança.* (MRNPT 12)

#### Desempenho de papéis

O desempenho de papel como mãe dentro da UTIN é demonstrado através de discursos em vários sentidos, observando-se que os significados mudam de acordo com o momento vivenciado.

*Tá sendo beem* [...] *assim, coisa nova, diferente... porque eu imaginei de outra forma, que eu ia dá mama, né, na boquinha dela. Aí eu tenho que tiráá, ir lá pro Banco de Leite, que lá eles trazem, dão na sonda. Aí é bem diferente do jeito que eu imaginava.* (MRNPT 10)

*Eu venho todo dia, mas* [...] *tá sendo boa. Eu já, já troquei, já, já pego neles.* (MRNPT 14)

*Eu, às, às vezes, eu fico meio perdida, mas as meninas sempre me ajudam* [equipe de enfermagem] *quando eu tenho alguma dúvida* [...]*. Eu tô muito ansiosa já dele ficar no meu colo, essas coisas assim. Aí eu fico meio agoniadinha já.* (MRNPT 09)

*Eu tô tentando dar o melhor, né. Tô tentando ser uma, uma mãe presente pra ele, que ele sinta minha presença ali, né. É* [...] *e eu quero passar essa tranquilidade pra ele também, né.* (MRNPT 05)

#### Interdependência

No cenário vivenciado pela mãe, observa-se que ela faz questão de mudar toda a sua vida para estar o máximo de tempo possível ao lado de seu filho. Contudo, caso ela não se sinta bem acolhida, sua permanência pode ser alterada, o que gera situações opostas ao que propõe as diretrizes do MC para sua primeira etapa. Isso pode impactar a criação de vínculo entre o binômio, nos cuidados prestados por ela e o preparo para uma alta segura, que envolve um processo minucioso trabalhado pela equipe da UTIN com essa mãe^(22)^.

*Todo mundo, minha família, a família do meu marido, tudo, meus amigos, os profissionais, que eu passei um bom tempo aqui, e eu já tô num ambiente que eu conheço, assim, mais um pouco de pessoa. Até eles mesmo* [profissionais da UTIN] *me dão força.* (MRNPT 13)

*Ela* [enfermeira] *me orientou a lavar minhas mãos quando eu chego, colocar touca, não pode entrar com o cabe. Com nenhum acessório, colocar máscara, e ao tocar na neném a gente tem que passar bastante álcool em gel pra ter todo um cuidado, porque a maturidade é* [...] *é* [...] *a prematuridade extrema, ela requer um pouquinho mais de* [...] *um pouco mais de cuidado.* (MRNPT 03)

*Sim, tô sendo cuidada sim. É, é como lhe falei, eu, é, é bom, ser orientada, eu fico mais tranquila, mais* [...] *eu fico mais informada, e isso, isso me dá um pouco de alívio nos pensamentos ruins, né. Eu* [...] *eu me sinto mais aliviada.* (MRNPT 08)

*No momento tô recebendo só auxílio sobre as meninas, né. Só sobre elas, a recuperação delas e os cuidados com elas.* (MRNPT 12)

Observa-se, a partir das falas das participantes, que, no parto prematuro, assim como nasce o RN antes do período adequado, também nasce uma mãe prematura, cheia de incertezas, sentimentos conflitantes e traumas, pois rompe com um momento único de construção e desenvolvimento pessoal, a partir de um evento repentino, inesperado^(6)^. Todavia, mesmo que o cenário costume confrontar a pessoa de forma imediata, a forma como a pessoa responde aos estímulos pode ser diferente. E como o cenário pode sofrer mudança, seja de forma repentina ou ao longo de um período, a forma de reagir aos estímulos também pode sofrer alteração, pois eles podem confrontar a pessoa de maneira mais perspicaz, ficando as respostas mais evidentes^(13)^.

## DISCUSSÃO

As falas das mães que relatam como está sendo enfrentar o diagnóstico da prematuridade atualmente evidenciam os estímulos que estão presentes. Percebem-se algumas mudanças em relação aos sentimentos, que apesar de ainda expressarem preocupação, apreensão e ansiedade, também dão lugar a sentimentos de tranquilidade, força e alegria por cada conquista. O toque e o contato pele a pele também se mostraram um estímulo positivo, estreitando o vínculo entre o binômio e influenciando a mãe a ter emoções positivas^(8)^.

Portanto, sentimentos de esperança tomam forma e se tornam mais evidentes, assim como a certeza de vivenciar um dia de cada vez, como uma forma de se preparar caso haja alguma mudança na saúde de seu filho, já que a prematuridade remete a um quadro clínico delicado, que pode sofrer mudanças repentinamente. Devido a isso, a prematuridade continua sendo o cenário principal em que essas mães se encontram. Assim, o estímulo focal está relacionado à quebra do que foi idealizado no momento do nascimento, ao diagnóstico de prematuridade do filho e aos sentimentos envolvidos ao redor desse diagnóstico^(13)^.

Acerca do estímulo contextual, o ambiente da UTIN pode ser bastante desafiador para essas mães, sendo o primeiro impacto causado pelo desconhecimento acerca de sua estrutura e seu funcionamento. Devido a isso, o Ministério da Saúde, através do MC, enfatiza a importância de os pais conhecerem a maternidade onde planejam que ocorra o nascimento do seu filho, além de saber a estrutura disponível, que inclui a UTIN, assim como participar de palestras com profissionais e famílias que passaram por essa experiência de internação na unidade. Essa recomendação tem o propósito de que os pais se sintam mais seguros, com melhor aceitação, caso seja necessária a internação de seu filho após o nascimento e, consequentemente, diminuir a ansiedade^(17-23)^.

Conforme salientado também pelo MC, a presença de uma rede de apoio com afeto, amparo e empatia em relação à situação da mãe remete a um significado de proteção em relação à separação física entre ela, seu filho e sua família, além de oferecer apoio emocional para ajudá-la a enfrentar esse momento difícil de mudanças. Além disso, contribui para que ela desenvolva o seu papel materno de forma mais efetiva^(8)^.

Observou-se que os estímulos residuais apresentados pelas participantes influenciaram inicialmente o seu comportamento, mesmo que inconscientemente. Contudo, tais estímulos sofreram modificações com as vivências atuais dessas mulheres, modificações essas desencadeadas pelo estímulo focal (mudança na percepção acerca do diagnóstico de prematuridade do filho atual) e contextual (ambiente físico e relacional na UTIN e presença de rede de apoio), que estimularam um quadro de adaptação favorável^(13)^.

A resposta no modo função fisiológica se associa a manifestações físicas e fisiológicas do organismo, podendo alterar também o comportamento emocional da pessoa. Experimentar sentimentos negativos em relação à prematuridade e internação de seu filho em uma UTIN é quase que inevitável para essa mãe. O período do puerpério, que já apresenta alterações na vida dessa mãe, naturalmente acaba tendo um comprometimento além do esperado, visto que há alteração no seu padrão de sono e repouso, devido ao seu deslocamento frequente até a UTIN para acompanhar seu filho. Dessa forma, a puérpera, que deveria ter um período de repouso para se recuperar de todo o processo do pré-parto, parto e pós-parto, seja em pós cesárea - em que a recuperação é mais demorada - ou em pós-parto via vaginal, passa a não ver sua saúde como prioridade naquele momento, vivenciando um cansaço maior que o habitual^(11,13)^.

Não obstante, é indicado que os profissionais envolvidos na assistência, particularmente os da área da enfermagem - que acompanham o binômio mãe e filho de forma contínua -, sempre considerem e fiquem atentos ao quadro de respostas apresentado pela mãe, pois, dessa forma, poderão intervir através de ações com o objetivo de reduzir as respostas desfavoráveis que ela possa apresentar^(14-24)^. Considerando o contexto do MC, também é fundamental que o profissional de saúde possibilite o contato entre mãe e filho o mais precocemente possível, pois essa influência tem demonstrado resultados positivos na estabilização dos sintomas fisiológicos tanto da mãe quanto do bebê, na redução dos níveis de estresse materno e no aumento do vínculo entre eles^(8-24)^.

No modo autoconceito, que trata da necessidade básica de integridade psíquica da pessoa, referindo-se aos seus aspectos psicológicos e espirituais, o apego à espiritualidade teve destaque como fonte de força para essas mães. Também é importante ressaltar que problemas de adaptação nesse modo podem interferir na capacidade de cura da pessoa ou mesmo para que ela realize os cuidados mínimos para a manutenção de sua saúde^(13)^. Em estudo desenvolvido com seis mães de RNPTs internados em um Hospital Amigo da Criança, as participantes relataram que o nascimento abrupto de seus filhos e posterior internação na UTIN prejudicou, de alguma forma, a construção psíquica acerca desse novo olhar sobre si, da maternidade e de seu filho^(25)^.

O desempenho de papel está relacionado diretamente à presença física dessa mãe na UTIN, visto que, segundo o MC, a mãe não é visita, mas sim acompanhante de seu bebê, mesmo que este se encontre em cuidados intensivos^(8)^. Esse modo gira em torno do que a pessoa tem ou almeja para si. A adaptação a essa nova função irá depender de como ela irá encarar as mudanças diante de si, refletido no quadro de prematuridade e no ambiente da UTIN, que pode levar a um risco dessa mãe se distanciar dos cuidados com o seu filho, culminando em um cenário onde esses cuidados acabam sendo prestados em sua totalidade pelo profissional de saúde. Por essa razão, é essencial que esse profissional auxilie e oriente essa mãe acerca dos cuidados com o RN e a reconhecer sinais de alerta, tendo em vista que será ela quem cuidará dele no momento posterior à hospitalização^(5,24)^.

A execução de atividades rotineiras, como carregar o filho no colo e o ato de amamentá-lo, que são características naturais do papel materno, acabam sofrendo alterações, levando à obtenção do papel materno de forma tardia, resultante da limitação de contato e interação entre mãe e filho^(11)^. Em estudo desenvolvido em UTINs de quatro estados brasileiros, dez mulheres que tiveram parto prematuro foram entrevistadas acerca de sua experiência na primeira semana de hospitalização do RN. Foram apresentados relatos de medo para realizar o contato físico com seu RN, pois o percebia como um ser frágil e muito pequeno, rodeado de dispositivos (tubo orotraqueal, sonda orogástrica, cateter central, entre outros), e o manuseio poderia causar a desconexão de algum desses, representando uma barreira para que esse contato acontecesse e limitando também os cuidados essenciais, como a troca de fralda^(26)^.

Todavia, ao longo da internação, as mães presentes na UTIN do estudo começam a perceber as diversas maneiras de prestar cuidados aos seus filhos, considerando suas especificidades, e de se aproximar e entrar em contato com eles, utilizando, por exemplo, a posição canguru, por meio do aprendizado diário^(24)^.

Na interdependência, os sistemas de apoio se configuram através das relações interpessoais, acontecendo habitualmente com pessoas próximas ao indivíduo, em que as necessidades afetivas são satisfeitas em um cenário onde a pessoa que cuida também é cuidada^(14)^. Em estudo realizado, as mães abordadas mencionam que se sentem cuidadas pela equipe da UTIN, assim como observam o esforço no cuidado com seus filhos, o que colabora com a evolução clínica do neonato. Também fica clara a importância em instruí-las a respeito dos equipamentos que rodeiam o RN, pois são essenciais para sua sobrevivência, mas sem a adequada explicação sobre sua finalidade e funcionamento, acabam representando barreiras para o contato entre a díade^(5)^.

Considerando que Roy refere que o presente modo possui a sensação de segurança como principal necessidade básica de alimentar os diversos tipos de relacionamentos, a adequação eficaz dá-se a partir das relações com essa natureza, que, como resultado, estimulam outros sentimentos positivos, que possam contribuir com a adaptação da mãe à sua realidade^(13)^. Destarte, as relações interpessoais, a depender do contexto e de seu progresso, podem facilitar ou dificultar o processo de adaptação. A presença do diálogo e sua clareza por parte do profissional de saúde com a mãe na UTIN remete a uma relação benéfica, em que a última sente segurança, acolhimento, respeito e valorização como resultado dessa interação^(5,11)^.

### Limitações do estudo

A limitação do estudo refere-se a ter sido realizado com mães de RNPTs internados nas UTINs de um mesmo serviço de saúde, pois tal fato pode ter contribuído para que houvesse similaridades nas vivências das participantes, sendo que a realização do estudo em outras instituições de saúde pode levar a uma maior possibilidade de encontrar diferentes experiências e discursos.

### Contribuições para a área da enfermagem, saúde ou políticas públicas

O presente estudo poderá contribuir com a assistência prestada pelo enfermeiro na promoção da adaptação das mães de RNPTs durante a internação na UTIN, a partir do conhecimento das experiências vivenciadas por essas mães, no intuito de minimizar a ansiedade e as preocupações que elas possam apresentar, assim como o medo acerca do desconhecido por conta da hospitalização de seu filho logo ao nascimento. Também poderá incentivar estudos futuros a respeito do assunto.

## CONSIDERAÇÕES FINAIS

Foi possível concluir que as participantes apresentaram, após um período de internação do seu filho, adaptação eficaz. Contudo, conforme preconiza a primeira etapa do MC, tal adaptação precisa ser o mais precoce possível. Inferiu-se que as respostas comportamentais ineficazes observadas estão ligadas, em sua maioria, à ausência de cuidados com essa mãe, principalmente pelos profissionais de saúde da UTIN, que são atores fundamentais para garantir assistência integral e qualificada segundo a política do MC, assim como no processo de adaptação dessas mães.

A utilização da Teoria de Roy permitiu reconhecer que os cuidados de enfermagem devem abranger também a mãe, que nem sempre é priorizada, mas precisa se adaptar a uma realidade até então totalmente desconhecida para ela, carregada de medo e insegurança com o futuro do filho, podendo levá-la a apresentar uma resposta positiva ou negativa. É importante que o enfermeiro realize constantemente o ato de avaliar e intervir junto a essa mãe como forma de alterar os estímulos que possam prejudicar a sua integridade e, consequentemente, sua adaptação ao meio ambiente.

## Data Availability

Os dados de pesquisa estão disponíveis no corpo do artigo.
